# Supplementary material for: Agave Fructans as a Carbon Source to Develop a Postbiotic-Based Strategy for the Prophylaxis and Treatment of Helicobacter pylori Infection
Source: Int J Mol Sci. 2025 Nov 17;26(22):11119. doi: 10.3390/ijms262211119 (PMC12652299; doi:10.3390/ijms262211119)
Supplement: Supplementary file 1 [file ijms-26-11119-s001.zip › ijms-3948278-supplementary.pdf]

Table S1. Overview of Pathogenicity-Related and Antibiotic Resistance Features in *H. pylori* Reference Strains

Feature	*H. pylori* 26695	*H. pylori* J99	*H. pylori* ATCC 43504
**Origin and usage**	Isolated from a gastritis patient; reference strain for genomics [65]	Isolated from a duodenal ulcer patient; used in genomic plasticity studies [66]	Type strain (ATCC/NCTC); isolated from gastric adenocarcinoma; infects gerbils; used as control strain [67]
**Genome size / CDSs**	1,667,867 bp; ~1,590 coding sequences [65]	1,643,831 bp; ~1,496 coding sequences [66]	1,680,829 bp; 1,615 coding sequences [67]
**cagPAI (pathogenicity island)**	Present; complete ~28–30 gene island including *cagA* and functional T4SS [68,71]	Present; similar to 26695 with variations in *cagI*/*cagN* [66,69]	Present and complete; *cagA* western type (~3.7 kb); functional T4SS [67]
**Key virulence factors**	*cagA*$^{+}$, *vacA* s1/m1, *babA*, *sabA*, *oipA*, *hopQ*, *ureA/B*, *katA* [65,68,70]	*cagA*$^{+}$, *vacA* s1/m1, *babA*, *sabA*, *oipA*, *hopQ*, *katA*; minor genetic variations [66,69,72]	*cagA*$^{+}$, *vacA* s1/m1, two *babA* copies, *sabA*, *hopZ*, *ureA/B*, *katA*; 68–85 virulence genes identified [67,73]
**Antibiotic resistance genes/mutations**	*rdxA*, *frxA* mutations → metronidazole; 23S rRNA A2142G/A2143G → clarithromycin; *gyrA* mutations → levofloxacin [74,75]	Variable resistance; similar mutations observed in clinical isolates but less characterized [76]	*rdxA*, *frxA* (deletions/IS605); *gyrA* (N87K/D91N); *cmeABC/cmeDEF* efflux systems; 23S rRNA mutations [67]

Figure S1. Growth of *H. pylori* 43504 by EPSs from INP_MX_001 LAB strain grown with GTFA from EPSs extract at 9 mg/mL.

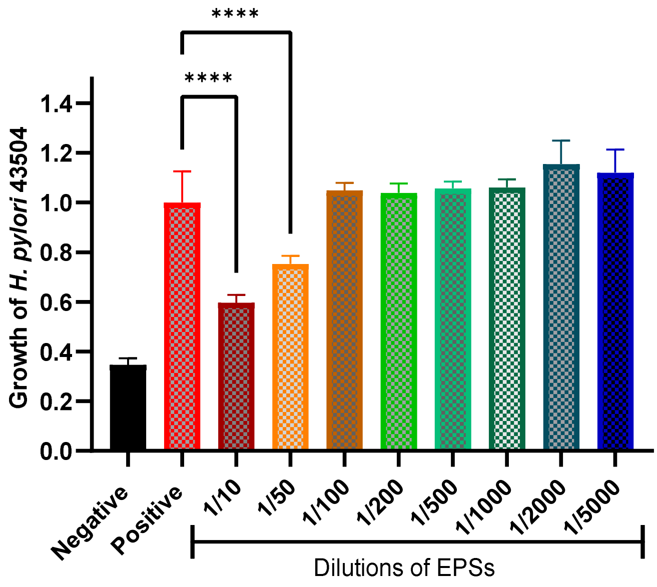

Supplementary material. Therapeutic treatment with EPSs using GTFA as the carbon source against *H. pylori* 43504. The *Helicobacter pylori* inoculum used in this treatment was 10^9 CFU/mL, equivalent to McFarland 4. Dilutions ranging from 10 to 5000 were prepared from an EPSs extract obtained from the growth of the INP_MX_01 LAB strain with GTFA as the sole carbon source. The results are plotted as the mean ± Std. deviation; a $p < 0.05$ was considered significant (****$p < 0.0001$).
